# Impact of the red blood cell distribution width-to-albumin ratio on neurological outcomes in cardiac arrest patients

**DOI:** 10.3389/fneur.2026.1732315

**Published:** 2026-09-04

**Authors:** Xiaoli Yang, Haiyan Wang, Fei Wang, Fei Peng

**Affiliations:** 1 Department of Anesthesiology, The Third People’s Hospital of Chengdu, Chengdu, China; 2 Department of Anesthesiology, Sichuan Provincial People's Hospital, University of Electronic Science and Technology of China, Chengdu, China

**Keywords:** brain injury, cardiac arrest, ischemia/reperfusion, neurological outcomes, red blood cell distribution width-albumin ratio

## Abstract

**Objectives:**

Brain injury remains the leading cause of mortality and morbidity among patients who experience cardiac arrest and cardiopulmonary resuscitation (CA/CPR). There is an urgent need to explore simpler and cheaper biomarkers to predict post-CA neurological outcomes. The red blood cell distribution width-to-albumin ratio (RA) has been used to assess inflammatory responses and prognosis in several diseases. This study was conducted to assess the correlation between RA levels and post-CA neurological outcomes, and to determine the value of RA in predicting neurological function after CA/CPR.

**Methods:**

We conducted a retrospective observational study on out-of-hospital cardiac arrest (OHCA) cases presenting to our hospital’s emergency department between January 2023 and April 2026. All patients were divided into cohorts with good or poor neurological outcomes according to cerebral performance categories (CPCs). Cox regression models were used to estimate the correlation between RA and neurological outcomes. The area under the receiver operating characteristic curve (AUC) was calculated to evaluate the ability of RA to predict neurological outcomes in patients after CA/CPR.

**Results:**

In total, 80 patients were involved, of whom 10 survived to hospital discharge with good neurological function (CPC ≤ 2), while the remaining subjects showed poor neurological function (CPC ≥ 3). RA levels were significantly higher in patients with poor neurological outcomes than in those with good outcomes. The AUC for the RA was 0.90, indicating excellent predictive accuracy. The RA values were independently associated with poor neurological outcomes following CA/CPR, even after adjusting for potential confounding factors.

**Conclusion:**

We found that RA is associated with post-CA neurological outcomes, and higher RA levels could serve as a predictor of worse neurological outcomes in patients who undergo CPR after experiencing CA.

## Introduction

Although the early initiation of high-quality cardiopulmonary resuscitation (CPR) increases the odds of desirable neurological outcomes among cardiac arrest (CA) patients, there are still more than 60% of CA patients who die from post-cardiac arrest brain injury (PCABI) (1–3). PCABI continues to remain the leading cause of mortality and morbidity among CA survivors (4). Currently, the clinical assessment of neurological outcomes of CA patients relies primarily on neurological examinations and neuroimaging (5, 6). However, due to the high incidence and severity of PCABI, simpler and cheaper biomarkers to predict the neurological outcomes of CA patients are urgently needed.

The key mechanisms underlying PCABI are ischemia and reperfusion injury, which occur sequentially during CA/CPR and the post-resuscitation phase. The main components of reperfusion injury are the activation of the innate immune system and subsequent neuroinflammation (7). Red blood cell (RBC) distribution width (RDW) reflects erythrocyte homeostasis and is widely used as a biomarker for systemic inflammation. The larger the RDW value, the more severe the imbalance of RBC homeostasis and the inflammatory response (8, 9).

Albumin (ALB), the most abundant protein in plasma, is synthesized in the hepatic tissue. In addition to reflecting nutritional status, it also has neuroprotective properties through its anti-inflammatory effects and inhibition of neuronal apoptosis (10, 11). More importantly, previous studies have reported that low serum ALB levels are associated with poor neurological outcomes after brain ischemia and reperfusion injury (10, 12).

It has been reported that RA, a metric derived by dividing RDW by ALB, is associated with the prognosis of several cardiovascular and cerebrovascular diseases, including stroke, carotid artery plaque, and acute myocardial infarction (9, 10, 13, 14). Nevertheless, the impact of RA on the neurological outcomes of CA/CPR patients remains largely unexplored. Consequently, our study aimed to collect data from our institution’s medical records to investigate the association between RA levels and neurological outcomes in CA/CPR patients. This updated understanding of RA could influence treatment strategies for CA/CPR patients and improve neurological outcomes after CA/CPR.

## Materials and methods

### Description of the study

This is a single-center, retrospective observational study conducted at Sichuan Provincial People’s Hospital, affiliated with the University of Electronic Science and Technology of China. The study protocol was reviewed and approved by the Ethics Committee of Sichuan Provincial People’s Hospital (No.785). Since this is a retrospective observational study and no care interventions were involved, written informed consent was waived by the Ethics Committee. We enrolled all out-of-hospital cardiac arrest (OHCA) cases that presented to the emergency department of our hospital between 1 January 2023 and April 2026, as confirmed by the absence of pulse, unresponsiveness, and apnea. Blood samples were collected upon arrival. We excluded patients younger than 18 years old, those with missing data in their medical records, and those who did not achieve return of spontaneous circulation (ROSC). We followed the Strengthening the Reporting of Observational Studies in Epidemiology (STROBE) checklist throughout the investigation (Supplementary materials).

### Study variables

The following study variables were collected from medical records, which included patients’ demographic characteristics, medical history, CA/CPR details, cerebral gray and white matter ratio (GWR), survival, cerebral performance categories (CPCs), and laboratory results, including white blood cells (WBCs), neutrophil granulocytes (NEUT), hemoglobin (HGB), platelets (PLTs), alanine aminotransferase (ALT), aspartate aminotransferase (AST), blood creatinine (CR), RDW, ALB, total cholesterol (TC), high-density lipoprotein cholesterol (HDL), brain natriuretic peptide (BNP), and glycosylated hemoglobin (HbA1c).

### Assessment of neurological outcomes

It has been reported that a loss of distinction between gray matter (GM) and white matter (WM) on computed tomography (CT) scans is an indicator of poor neurological outcomes among CA survivors (6, 15). According to our previously published method (16), the gray-to-white matter signal intensity ratio (GWR) was analyzed using brain CT images that were taken within 8 h after ROSC. Additionally, the cerebral performance categories (CPCs) were used to assess neurological outcomes at hospital discharge. CPC 1 and CPC 2 were considered as good cerebral performance. CPC 3–5 were identified as poor cerebral performance (17).

### Statistical analysis

Once data collection and verification were completed and the database was finalized, all data were analyzed independently by two authors using SPSS version 25.0 (SPSS Inc., Chicago, IL, USA), with any discrepancies resolved by a third author. Quantitative variables were described as the mean±SD, while qualitative variables were expressed as counts and percentages. Continuous variables were compared with a two-tailed Student’s t-test. A chi-square test was used to compare the two groups for qualitative variables. Cox regression models were used for estimating the relationship between RA and neurological outcomes; the results were presented as odds ratios (ORs) with 95% confidence intervals (CIs). Covariates in model 1 were adjusted for time to basic life support, while covariates in model 2 were adjusted for time to ROSC, and covariates in model 3 were adjusted for age. A *p*-value of less than 0.05 was considered statistically significant.

## Results

### Study characteristics

In this study, 80 patients were eventually included to evaluate the association between RA and neurological outcomes after CA/CPR. Of these 80 patients, 10 survived to hospital discharge with good neurological function (CPC ≤ 2), and the remaining showed poor neurological function (CPC ≥ 3). The baseline characteristics of these patients are summarized in Table 1. Patients in the poor neurological function group were older, were more likely to have experienced cardiac arrest at home, and were likely to have had delayed time to ROSC and time to basic life support. Additionally, in the poor neurological function group, more patients were intubated. Regarding laboratory findings, patients in the poor neurological function group had higher levels of RDW, CRP, and BNP than those with good neurological function after CA/CPR.

**Table 1:** Baseline characteristics of the study population according to neurological outcomes.

Items	Good (CPC ≤ 2) (*n* = 10)	Poor (CPC ≥ 3) (*n* = 70)	*p*
Demographic characteristics			
Age, mean±SD, years	55 ± 11	65 ± 10	0.014
Sex, male (%)	4(40.0)	35(50.0)	0.364
Weight, mean±SD, kg	66 ± 13	71 ± 14	0.577
Medical history			
Hypertension, *n* (%)	4(40.0)	25(35.7)	0.636
Chronic heart diseases, *n* (%)	3(30.0)	12(17.1)	0.554
Diabetes, *n* (%)	3(30.0)	25(35.7)	0.821
Chronic respiratory diseases, *n* (%)	2(20.0)	8(11.4)	0.805
Smokers, *n* (%)	4(40.0)	35(50.0)	0.509
CA/CPR location			0.039
Home, *n* (%)	4(40.0)	43(61.4)	
Public, *n* (%)	6(60.0)	27(38.6)	
CA/CPR details			
Time to basic life support, mean±SD, minutes	2.1 ± 1.5	5.2 ± 2.8	0.039
Time to advanced life support, mean±SD, minutes	6.3 ± 2.3	8.5 ± 3.3	0.365
Time to ROSC, mean±SD, minutes	15.4 ± 6.4	20.1 ± 8.5	0.033
Intubation, *n* (%)	5(50.0)	49(70.0)	0.049
WBC (10^9^/L)	12.5 ± 2.2	13.8 ± 2.9	0.525
NEUT (10^9^/L)	11.2 ± 2.5	11.9 ± 3.7	0.884
HGB (g/L)	126.1 ± 21.7	125.7 ± 26.4	0.732
PLT (10^9^/L)	130.4 ± 31.3	130.3 ± 29.8	0.794
ALT (U/L)	45.5 ± 20.9	50.5 ± 26.9	0.232
AST (U/L)	51.3 ± 21.9	52.6 ± 20.9	0.494
CR (μmol/L)	88.0 ± 21.3	87.0 ± 23.7	0.605
RDW (%)	13.7 ± 2.2	18.1 ± 3.9	0.038
ALB (g/dL)	3.7 ± 0.5	2.9 ± 0.4	0.027
TC (mmol/L)	4.7 ± 1.3	4.8 ± 1.2	0.892
HDL (mmol/L)	0.9 ± 0.5	0.8 ± 0.5	0.579
HbA1c (%)	4.6 ± 1.9	4.7 ± 2.5	0.346
CRP (mg/L)	20.5 ± 11.9	29.9 ± 17.7	0.010
BNP (pg/mL)	402.1 ± 38.1	563.0 ± 49.9	0.014

ROSC, return of spontaneous circulation; WBC, white blood cell; NEUT, neutrophil; HGB, hemoglobin; PLT, platelet; ALT, alanine transaminase; AST, aspartate aminotransferase; CR, creatinine; RDW, blood cell distribution width; ALB, albumin; TC, total cholesterol; HDL, high-density lipoprotein; HbA1c, glycated hemoglobin; CRP, C reactive protein; BNP, brain natriuretic peptide.

### Association between RA and neurological outcomes after CA/CPR

**Figure 1:**
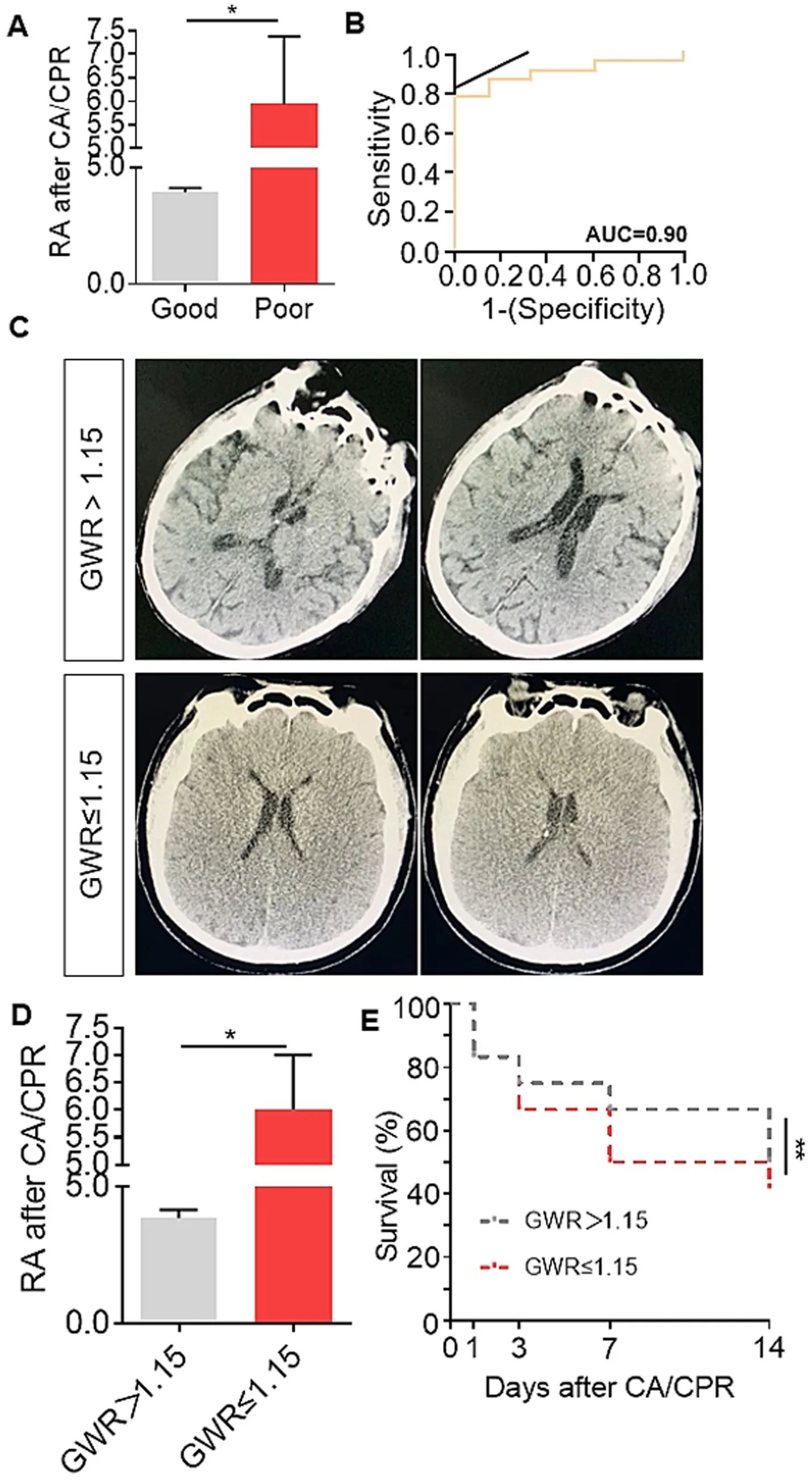
Association between RA and neurological outcomes after CA/CPR. **(A)** RA levels in patients in the good neurological outcome group and the poor neurological outcome group. **(B)** Predictive accuracy of RA for neurological outcomes after CA/CPR. **(C)** Representative brain computed tomography (CT) images of patients after CA/CPR. **(D)** RA levels of patients with GWR > 1.15 and GWR ≤ 1.15. **(E)** Survival rate of patients with different GWRs. **(F)** *p < 0.05, **p < 0.01.

We found that RA levels were significantly higher in patients with poor neurological outcomes than in those with good outcomes (3.98 ± 0.84 vs. 5.89 ± 1.24, *p* = 0.028; Figure 1A). The area under the receiver operating characteristic curve (AUC) of RA for their neurological outcomes was 0.90 (0.79–0.99), indicating excellent predictive accuracy (Figure 1B) (18). At the cutoff point of 4.5, the predictive accuracy of RA for poor neurological outcomes demonstrated a sensitivity of 1.00 (1.00–1.00) and a specificity of 0.80 (0.64–0.91). Notably, all 10 patients with RA levels below 4.5 showed good neurological outcomes at the time of hospital discharge. Additionally, we found that the AUCs for RDW and serum albumin were 0.79 and 0.70, respectively (Supplementary Figures S1A,B). When comparing AUCs, RA proved to be a better predictor of neurological outcomes after CA/CPR than RDW and serum albumin.

The relationship between RA and neurological outcomes could be more directly elucidated by examining the association between the GWR and RA. Therefore, we divided all patients into two groups based on the GWR: A GWR of ≤1.15 indicated a poor neurological outcome with significantly altered neuronal structure after CA/CPR, while a GWR of >1.15 indicated a good neurological outcome with minimal or moderately altered neuronal structure after CA/CPR (Figure 1C). We found that RA levels were significantly higher in patients with a GWR ≤ 1.15 than in those with a GWR > 1.15 (3.88 ± 0.76 vs. 6.12 ± 1.13, *p* = 0.021; Figure 1D). Consistently, lower survival rates were also observed in patients with a GWR ≤ 1.15 than in those with a GWR > 1.15 (Figure 1E).

### Logistic regression analyses

The association between RA and neurological outcomes was further verified by adjusting for several confounding factors. We found that the time to basic life support, the time to ROSC, and the age of patients were significantly different between patients with good and poor neurological outcomes. Therefore, we further evaluated the influence of time to basic life support, time to ROSC, and the age of patients on the association between RA levels and neurological outcomes. We performed three separate multivariate logistic regression analyses (model 1 was adjusted for time to basic life support, model 2 was adjusted for time to ROSC, and model 3 was adjusted for age). As shown in Table 2, the association between RA and neurological outcomes after CA/CPR persists, even after adjusting for the aforementioned confounding factors.

**Table 2:** Logistic regression analyses.

Variables	OR (95%CI)	*p*
Univariate		
RA, (%)/(g/dL)	1.342 (1.077, 1.821)	0.019
Multivariate (model 1)		
RA, (%)/(g/dL)	1.469 (1.172, 1.854)	<0.001
Time to basic life support, minutes	0.837 (0.704, 0.985)	0.019
Multivariate (model 2)		
RA, (%)/(g/dL)	1.295 (1.003, 1.629)	0.031
Time to ROSC, minutes	0.851 (0.657, 0.962)	0.022
Multivariate (model 3)		
RA, (%)/(g/dL)	1.364 (1.110, 1.632)	<0.001
Age, years	0.839 (0.702, 0.994)	0.029

RA, red blood cell distribution width-albumin ratio.

## Discussion

RA can be easily derived from routine blood tests; however, the association between RA and post-CA neurological outcomes is poorly understood. In this cohort of OHCA patients, we established the correlation between RA and post-CA neurological outcomes. This is the first study that has shown that RA levels are significantly higher in CA/CPR patients with poor neurological outcomes than in those with good outcomes. In addition to these findings, the data suggest that RA might be a promising biomarker for predicting neurological outcomes after CA/CPR.

One published study explored the correlation between RA and the prognostic outcomes of patients with brain ischemia/reperfusion (I/R) injury (10). By encompassing 1,480 patients suffering from ischemic stroke for analysis, the findings demonstrated that RA may be an easily accessible and low-cost biomarker for predicting stroke-associated infections and mortality. Several studies have evaluated the association between RA and cardiovascular and cerebrovascular diseases. For instance, Liu et al. demonstrated that higher RA levels are associated with higher cardiovascular and all-cause mortality rates (9). Ni et al. revealed that elevated RA levels are positively associated with mortality rates among patients suffering from heart failure (19). Simultaneously, it was discovered that RA serves as a robust predictor of unfavorable outcomes among patients afflicted with coronary heart disease, acute myocardial infarction, and aneurysms (20–23). However, previous studies have only reported adverse outcomes, such as mortality and infection rates. Research on neurological outcomes was limited. Therefore, our study addressed this limitation and provided important insights into the association between RA and neurological outcomes after CA/CPR.

Oxidative stress and inflammation lead to the formation of immature red blood cells in the bloodstream, leading to inferior oxygen transport capabilities and potentially inducing hypoxia (24). Therefore, RDW has been reported to be closely related to the development of brain I/R injury, and a higher RDW could independently predict adverse outcomes in patients under such conditions (25, 26). Similarly, serum albumin levels have also been used to predict outcomes in patients with brain I/R injury. The lower the albumin level, the worse the prognosis of these patients (10, 12). To evaluate the ability of RA to predict the neurological outcomes of CA/CPR patients, we compared the AUCs for RA, RDW, and serum albumin. The results intuitively showed that RA was a better predictor than RDW and serum albumin alone. Additionally, RA can also be quickly derived from routine blood tests and is not based on other variables such as vital signs. Therefore, RA could be a simple and reliable parameter for risk stratification of CA/CPR patients.

Certain limitations of this study should be acknowledged. First, the conclusions are limited to a cohort of OHCA patients; the correlations between RA and the neurological outcomes of patients with in-hospital cardiac arrest are unexplored. Second, although this study can provide valuable insights, it has inherent limitations as a single-site cohort study. Therefore, multicenter studies with larger sample sizes are urgently needed in this field.

## Conclusion

In conclusion, in this cohort of OHCA patients, we found that RA is closely related to the post-CA neurological outcomes and that higher RA can independently predict poorer neurological outcomes in patients after CA/CPR.

## Data Availability

The raw data supporting the conclusions of this article will be made available by the authors, without undue reservation.
